# Variation in Patient Outcomes Across Nurse Practitioner Scope of Practice Levels

**DOI:** 10.1093/geroni/igaf122.4044

**Published:** 2025-12-31

**Authors:** Chih-Ying Li, Md Ibrahim Tahashilder, Letitia Graves, Peter Cram, Yong-Fang Kuo

**Affiliations:** University of Texas Medical Branch, Galveston, Texas, United States; University of Texas Medical Branch, Galveston, Texas, United States; University of Texas Medical Branch, Galveston, Texas, United States; Wright State University, Fairborn, Ohio, United States; University of Texas Medical Branch, Galveston, Galveston, Texas, United States

## Abstract

This study investigates whether varying degrees of authorized nursing practice patterns (restricted, reduced, full authority with transition, and full authority without transition) across the nation influenced patient outcomes (30-day hospital readmission and in-hospital mortality). The four practice patterns are defined based on the published annual legislative update for advanced practice registered nurses. Utilizing 2019 CMS Medicare administrative claims, we analyzed six medical conditions due to their high prevalence and burdens commonly targeted for quality improvement and policy: acute myocardial infarction (n = 153,870), heart failure (n = 449,232), pneumonia (n = 288,831), stroke (n = 250,291), total hip/knee arthroplasty (n = 538,283), and hip/femur fractures (n = 147,694). We conducted multivariable logistic regression, adjusting for age, sex, race/ethnicity, residential rurality, dual eligibility, Medicare original entitlement, Elixhauser comorbidity, hospital lengths of stay, hospital ownership, and median household income (at the zip-code level). Relative to the restricted practice group, patients in the reduced practice group were more likely to be readmitted (OR = 1.06, 95% CI = 1.04-1.08), whereas patients in both full authority groups demonstrated a reduced likelihood of readmission (with transition: OR = 0.91, 95% CI = 0.90-0.93; without transition: OR = 0.89, 95% CI = 0.88-0.91). Relative to the restricted practice group, patients in the reduced and full authority with transition groups had lower mortality risk (OR = 0.95, 95% CI = 0.93-0.98; OR = 0.99, 95% CI = 0.97-1.02, respectively), whereas patients in full authority without transition group demonstrated an increased mortality risk (OR = 1.11, 95% CI = 1.09-1.13). Our results suggest that the full nursing authority with transition produced relatively balanced and optimal results on both outcomes among Medicare beneficiaries.

